# Short- and long-term associations of insulin resistance indices with cardiovascular–kidney–metabolic syndrome risk: a retrospective cohort study

**DOI:** 10.3389/fendo.2026.1917797

**Published:** 2026-08-31

**Authors:** Hui Luo, Xueting Wang, Cheng Zhang, Jing He, Mengjie Sun, Yanlang Yang, Min Wang, Ruilei Hu, Shu Xiao, Zhonghui Chen, Changdong Shu, Xianhong Yin

**Affiliations:** 1 Department of Nephrology, The Affiliated Xuancheng Hospital of Wannan Medical University, Xuancheng, China; 2 Anhui Proof of Concept Center, Hefei, China; 3 Department of Nephrology, The First Affiliated Hospital of Wannan Medical University, Yijishan Hospital, Wuhu, China; 4 Department of Central Laboratory, The Affiliated Xuancheng Hospital of Wannan Medical University, Xuancheng, China

**Keywords:** cardiovascular–kidney–metabolic syndrome, insulin resistance, metabolic score for insulin resistance, risk predication, single point insulin sensitivity estimator, triglyceride–glucose–body mass index

## Abstract

**Background:**

Insulin resistance (IR) is a key driver of cardiovascular–kidney–metabolic (CKM) syndrome. This study evaluated the independent associations of three novel IR surrogate indices—the Metabolic Score for Insulin Resistance (METS–IR), Triglyceride–Glucose–Body Mass Index (TyG–BMI), and Single Point Insulin Sensitivity Estimator (SPISE)—with CKM syndrome risk and compared their predictive performance for short- and long-term outcomes.

**Methods:**

This retrospective cohort study included 12,334 Chinese adults aged ≥20 years enrolled between 2015 and 2019. Multivariable logistic regression and restricted cubic splines were used for association assessment, while area under the receiver operating characteristic curve (AUROC) analysis was used to evaluate predictive accuracy. Subgroup analyses were performed for age, sex, and BMI.

**Results:**

Analysis of 1- and 5-year follow-up outcomes revealed that the METS–IR and TyG–BMI were positively associated with CKM syndrome, whereas SPISE showed an inverse association (all *p* < 0.001). All indices exhibited nonlinear dose–response relationships. Adding each IR index separately to the base model improved discrimination: 1-year AUROC increased from 0.681 to 0.710–0.712 and 5-year AUROC from 0.719 to 0.737–0.741 (all *p* < 0.001). SPISE achieved the highest AUROC at both time points, pairwise comparisons with the other indices showed statistically significant differences of 0.002–0.003 (all *p* ≤ 0.033). Subgroup analyses indicated stronger associations in males and participants aged <40 years for short-term risk, with sex-specific effect modification for long-term outcomes.

**Conclusion:**

The METS-IR, TyG-BMI, and SPISE showed robust nonlinear associations with CKM syndrome risk. Their overall discriminatory performance was broadly comparable, with SPISE showing a marginal advantage. The associations were stronger in males, supporting their potential value in sex-tailored risk assessment.

## 1 Background

Cardiovascular–kidney–metabolic (CKM) syndrome has emerged as a major public health issue worldwide (1). The American Heart Association (AHA) defines CKM syndrome as a systemic disease caused by the pathological physiological interactions among metabolic disorders, chronic kidney disease, and cardiovascular diseases (CVDs) (2), leading to multiorgan dysfunction and even death (3–5). The incidence rate of CKM syndrome has risen continuously with an increase in unhealthy lifestyles and an aging population (1), posing a challenge to public health and economic development. It is estimated that the prevalence rate of comorbidities related to CKM syndrome is approximately 25%–30% worldwide (6). Among the Chinese population, the prevalence of CKM syndrome has increased 60.3% over the past decade (7). Therefore, there is an urgent need to identify reliable biomarkers for early identification of CKM syndrome risk, which is of vital importance for the prevention of CKM syndrome progression and effective clinical management.

Characterized by the reduced or impaired sensitivity of target organs or tissues to insulin (8), insulin resistance (IR) is widely recognized as a key upstream driver in the onset of CKM syndrome (9). As CKM syndrome develops, IR promotes its progression by fostering chronic inflammation, endothelial dysfunction, and metabolic disturbances (5, 10). Consequently, early identification of the insulin-resistant state is of great significance for identifying and predicting the early high-risk population for CKM syndrome. Owing to its high procedural cost and technical complexity, the euglycemic hyperinsulinemic clamp test, regarded as the gold standard for assessing IR, is currently unsuitable for large-scale population screening (11). Novel indicators for IR include the Metabolic Score for Insulin Resistance (METS–IR) (12), the Triglyceride–Glucose–Body Mass Index (TyG–BMI) (13), and the Single Point Insulin Sensitivity Estimator (SPISE) (14). As these three indicators can be measured much more cost-effectively using easily applicable direct assessment methods, they have been widely adopted in clinical research. METS-IR has been evaluated for incident type 2 diabetes in the Mexican population (15), the association was also observed in a rural Chinese cohort (16). Among individuals with chronic kidney disease (CKD) in the United States, TyG-BMI has been examined in relation to all-cause mortality (17). SPISE has been evaluated for identifying metabolic syndrome in North Indian adults (18), with similar predictive ability subsequently demonstrated in Korean adults (19). These indices have also been investigated for identifying IR among children and adolescents with obesity and for assessing stroke and cardiovascular disease risk among individuals with CKM syndrome (20–22). Although these studies demonstrate that METS-IR, TyG-BMI, and SPISE have been applied across diverse populations and a broad range of clinical conditions, no research has compared their predictive performance for CKM syndrome simultaneously.

Thus, this study was designed as a retrospective cohort study, aiming to evaluate the independent associations of METS-IR, TyG-BMI and SPISE with CKM syndrome, and further compare the risks of these three indicators for CKM syndrome in the short (1-year) and long (5-year) term.

## 2 Methods

### 2.1 Study design and population

**Figure 1:**
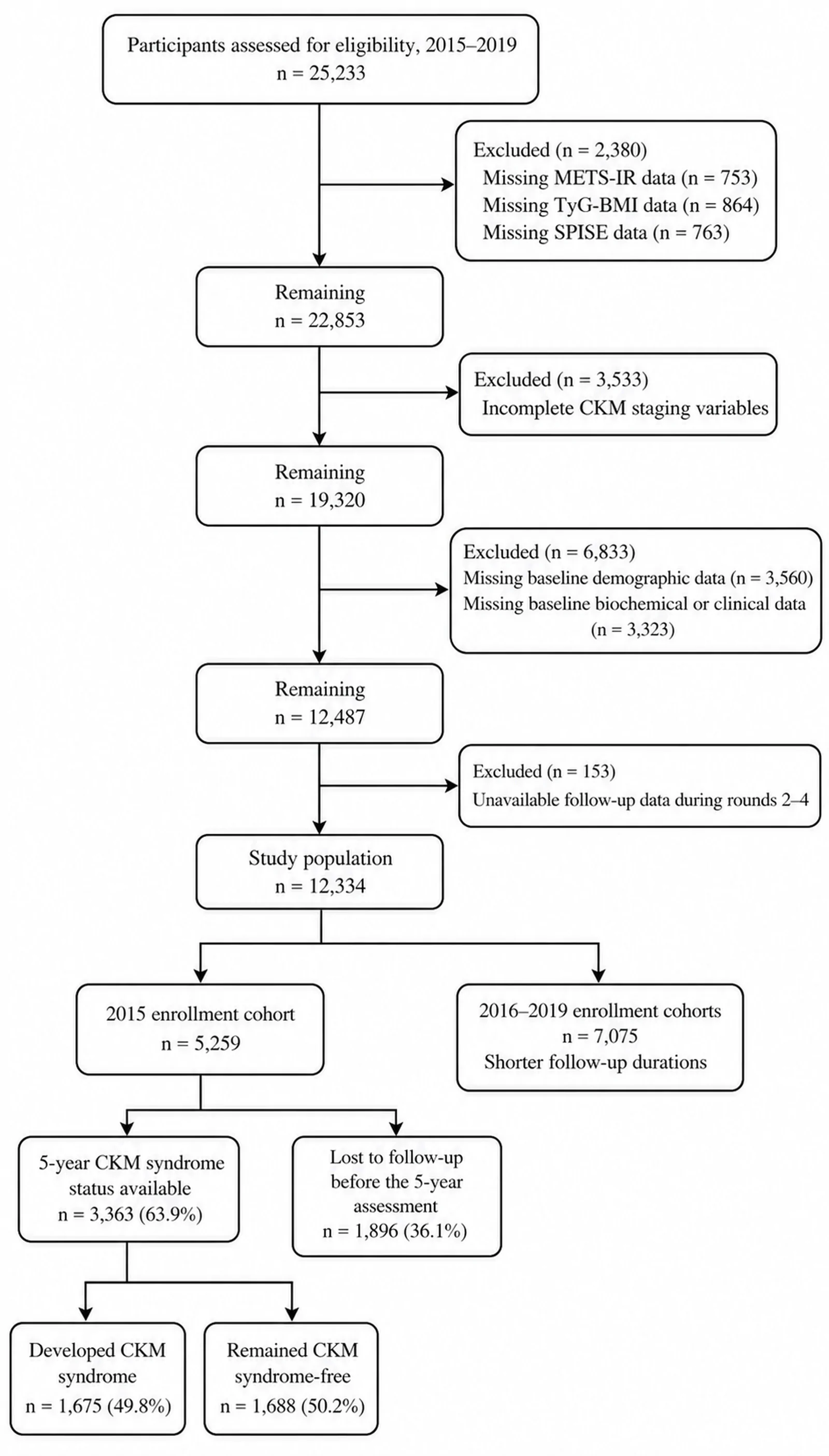
Flow diagram of participant selection.

This retrospective cohort study was a community-based long-term follow-up survey targeting the general population aged 20 and above in Anhui Province, China. The baseline survey conducted in 2015 was followed by four rounds of surveys: round 2 in 2016, round 3 in 2017, round 4 in 2018, and round 5 in 2019. The data from rounds 1 to 5 were utilized in this study. Participants were enrolled annually between 2015 and 2019, with five-year follow-up data available for the 2015 enrollment cohort. Baseline assessments were conducted from 2015 to 2019, and follow-up data were collected through 2020. The 2020 follow-up included the 1-year outcomes of participants enrolled in 2019 and the 5-year outcomes of those enrolled in 2015. In round 1, a total of 25,233 individuals were recruited. The inclusion criteria were as follows: (1) age 20 years or above and (2) completion of all follow-up data. The exclusion criteria were as follows: (1) lack of demographic, clinical, and/or biochemical data at baseline; (2) inability to calculate METS–IR, TyG–BMI, and/or SPISE; and (3) inability to accurately diagnose the stages of CKM syndrome. After 12,899 individuals were excluded, 12,334 participants were included and analyzed in the study (**Figure 1**). This study was conducted in accordance with the Declaration of Helsinki and was approved by the Institutional Ethics Committee of Xuancheng People’s Hospital (No. 2026-sjky10-01). All participants provided written informed consent.

### 2.2 Data collection

Baseline demographic, lifestyle, and clinical data were collected using self-report questionnaires. Blood pressure, height, and weight were measured by physicians, and blood samples were collected and analyzed by laboratory physicians. The baseline demographic and lifestyle information collected included age, sex, smoking status, and alcohol drinking status. The baseline clinical history collected included CVDs (cerebral infarction, stroke, cerebral hemorrhage, coronary heart disease, myocardial infarction, and heart failure), hypertension, diabetes mellitus, and hepatic disorders. The baseline clinical data collected consisted of systolic blood pressure (SBP), diastolic blood pressure (DBP), and body mass index (BMI). The baseline laboratory data collected were creatinine (CR), blood urea nitrogen (BUN), total cholesterol (TG), triglyceride (TG), high-density lipoprotein cholesterol (HDL-C), low-density lipoprotein cholesterol (LDL-C), and fasting blood glucose (FBG) levels.

The smoking status of the participants was divided into two categories: never or currently (“No” or “Yes”). Alcohol drinking status was divided into three categories: never, seldom, or frequently (“Never,” “Seldom,” or “Frequently”). SBP and DBP were obtained by standardized mercury sphygmomanometry after 5 min of seated rest. BMI was calculated as weight (kg)/height squared (m^2^). Weight and height were measured by trained physicians. After the participants had fasted continuously for at least 10 hours, blood samples were collected from their elbow veins. All laboratory measurements were conducted in a certified central laboratory following standardized quality control protocols.

### 2.3 Calculation of IR indices

Three surrogate indices of IR were calculated from baseline fasting laboratory and anthropometric data.

#### 2.3.1 METS–IR

The METS–IR was calculated according to the following formula (23):


$$\text{METS}–\text{IR}=\frac{\text{ln}[2\times \text{FBG}(\text{mg}/\text{dL})+\text{TG }(\text{mg}/\text{dL})]\times \text{BMI}(\text{kg}/{\text{m}}^{2})}{\text{ln}[\text{HDL}-\text{C}(\text{mg}/\text{dL})]}$$

#### 2.3.2 TyG–BMI

The TyG–BMI was computed according to the following formula (13):


$$\text{TyG}–\text{BMI}=\text{ln}[\text{TG}(\text{mg}/\text{dL})\times \text{FBG}(\text{mg}/\text{dL})/2]\times \text{BMI}(\text{kg}/{\text{m}}^{2})$$

#### 2.3.3 SPISE

The SPISE was calculated according to the following formula (24):


$$\text{SPISE}=\frac{\;600\times \text{HDL}-\text{C}{\left(\text{mg}/\text{dL}\right)}^{0.185}}{\text{TG}{\left(\text{mg}/\text{dL}\right)}^{0.2}\times \text{BMI}{\left(\text{kg}/{\text{m}}^{2}\right)}^{1.338}}$$

### 2.4 Definition and staging of CKM syndrome

CKM syndrome was staged according to the 2023 AHA scientific statement (2).Each participant was assigned to the highest stage for which the corresponding criteria were met. Overweight was defined as a BMI of 24.0–27.9 kg/m^2^ and obesity as a BMI ≥28.0 kg/m^2^ (25). Diabetes was defined as an FBG level ≥126 mg/dL or a self-reported history of diabetes. Hypertriglyceridemia was defined as a TG level ≥150 mg/dL. Hypertension was defined as an SBP ≥140 mmHg, a DBP ≥90 mmHg, or a self-reported history of hypertension. CKD was defined as an estimated glomerular filtration rate (eGFR) <60 mL/min/1.73 m^2^ or albuminuria. Metabolic risk factors included hypertriglyceridemia, hypertension, diabetes, and metabolic syndrome. Established clinical CVD was defined as a self-reported history of cerebral infarction, stroke, cerebral hemorrhage, coronary heart disease, myocardial infarction, or heart failure.

Based on the statement, the participants were classified into one of five stages: Stage 0: absence of overweight/obesity and metabolic risk factors; Stage 1: overweight/obesity, abdominal obesity, or dysfunctional adipose tissue without additional metabolic risk factors or CKD; Stage 2: presence of metabolic risk factors or CKD, or both; Stage 3: subclinical CVD. Risk equivalents for subclinical CVD encompasses very high-risk CKD (stage G4 or G5 CKD) and a high predicted 10-year CVD risk by the Framingham risk score (26). Stage 4: established clinical CVD. Participants at Stage 0 were classified as “CKM syndrome negative” and those at Stages 1–4 as “CKM syndrome positive.” This binary classification defined the primary outcome variable.

### 2.5 Statistical analysis

Baseline characteristics were summarized and stratified by enrollment year (2015–2019). Continuous variables were expressed as median (interquartile range [IQR]) or mean ± standard deviation (SD), as appropriate. Categorical variables were presented as n (%). Logistic regression models were fitted to assess the associations between insulin resistance indices and the risk of CKM syndrome. IR indices were standardized using Z-score transformation, and odds ratios (ORs) with 95% confidence intervals (CIs) were expressed per 1-standard-deviation (SD) increase. For categorical analyses, exposures were divided into quartiles (Q1–Q4), with Q1 serving as the reference. The linear trend across quartiles was evaluated by treating quartile rank as an ordinal variable. Restricted cubic splines (RCS) with 4 knots (5th, 35th, 65th and 95th percentiles) were applied to model potential non-linear relationships (see **Supplementary Methods** for details). Four nested models with progressive covariate adjustment were constructed. Sex, smoking status, alcohol consumption status, and hypertension status were treated as categorical variables. Age, SBP, DBP, lipid measurements (TC, TG, HDL-C, and LDL-C), and kidney function measurements (CR and BUN) were treated as continuous variables. Subgroup analyses were conducted to examine effect modifications by age, sex, and body mass index (BMI), with interaction effects tested using likelihood ratio tests (see **Supplementary Methods** for full details). The discriminatory performance of each predictive model was evaluated using the area under the receiver operating characteristic curve (AUROC), and the statistical significance of the difference between models was assessed using the DeLong test. The optimal cut-off values of METS-IR, TyG-BMI, and SPISE for predicting 1- and 5-year CKM syndrome outcomes were determined by maximizing the Youden index (sensitivity + specificity − 1). The corresponding sensitivity and specificity were calculated for each cut-off value. All analyses were conducted using R software (version 4.3.0), and two-sided *p* < 0.05 was considered statistically significant.

## 3 Results

The baseline characteristics of the 12,334 participants stratified by enrollment year (2015–2019) with CKM syndrome diagnosis outcomes during the 5-year follow-up period are shown in **Table 1**. The sample size varied across enrollment years, from 5,259 participants in 2015 to 256 in 2019. The median age ranged from 33 to 38 years, and females comprised the majority across all years (56.3%–69.0%). The cumulative incidence of CKM syndrome diagnosis at year 1 was comparable across enrollment years, ranging from 34.7% to 39.6%. Analysis of the complete 5-year follow-up data for the 2015 cycle revealed that the incidence of CKM syndrome diagnosis was 31.9% (n = 1675), with 32.1% (n = 1688) remaining CKM syndrome-free. The remaining 36.1% of the 2015 cohort participants (n = 1,896) were lost to follow-up. The IR indices remained relatively stable across enrollment years, suggesting consistent metabolic risk profiles among the participants. The mean TyG–BMI ranged from 177.52 ± 25.08 to 183.40 ± 25.65, the mean METS–IR ranged from 29.98 ± 4.53 to 30.77 ± 4.82, and the mean SPISE ranged from 6.87 ± 1.38 to 7.12 ± 1.45 across the years.

**Table 1:** Baseline characteristics of CKM disease-free participants during a five-year follow-up period.

Variable	2015	2016	2017	2018	2019
N	5259	2887	2096	1836	256
Age	38.00 (31.00, 48.00)	36.00 (29.00, 45.00)	34.00 (29.00, 44.00)	36.00 (29.00, 45.00)	33.00 (30.00, 40.00)
Sex (%)					
Male	2189 (41.6)	1154 (40.0)	650 (31.0)	803 (43.7)	96 (37.5)
Female	3070 (58.4)	1733 (60.0)	1446 (69.0)	1033 (56.3)	160 (62.5)
Smoking (%)					
No	4361 (82.9)	2440 (84.5)	1885 (89.9)	1575 (85.8)	225 (87.9)
Yes	898 (17.1)	447 (15.5)	211 (10.1)	261 (14.2)	31 (12.1)
Alcohol (%)					
Frequent	291 (5.5)	188 (6.5)	98 (4.7)	99 (5.4)	13 (5.1)
Seldom	746 (14.2)	318 (11.0)	139 (6.6)	170 (9.3)	9 (3.5)
No	4222 (80.3)	2381 (82.5)	1859 (88.7)	1567 (85.3)	234 (91.4)
BMI	21.92 (20.23, 23.81)	21.89 (20.26, 23.75)	21.68 (20.03, 23.59)	22.09 (20.36, 24.06)	22.30 (20.41, 23.90)
FGB, mmol/L	5.11 (4.81, 5.42)	4.81 (4.53, 5.13)	4.78 (4.51, 5.11)	4.77 (4.48, 5.09)	4.93 (4.63, 5.19)
TG, mmol/L	0.97 (0.75, 1.24)	0.86 (0.64, 1.13)	0.88 (0.66, 1.12)	0.92 (0.69, 1.20)	0.95 (0.72, 1.19)
TC, mmol/L	4.14 (3.68, 4.60)	4.11 (3.64, 4.54)	4.06 (3.63, 4.51)	4.18 (3.74, 4.59)	4.10 (3.71, 4.45)
CR, mmol/L	60.90 (52.00, 74.00)	62.60 (53.00, 76.85)	59.80 (52.10, 71.60)	62.20 (51.70, 73.90)	60.00 (50.88, 68.35)
BUN, mmol/L	5.07 (4.19, 6.07)	4.68 (3.87, 5.63)	4.50 (3.71, 5.37)	4.91 (4.13, 5.87)	4.90 (4.10, 5.68)
HDL-C, mmol/L	1.54 (1.34, 1.78)	1.44 (1.27, 1.63)	1.50 (1.32, 1.70)	1.47 (1.28, 1.68)	1.56 (1.38, 1.75)
LDL-C, mmol/L	2.08 (1.65, 2.51)	2.21 (1.83, 2.59)	2.12 (1.75, 2.47)	2.22 (1.88, 2.58)	2.10 (1.77, 2.41)
CKM diagnosis at year 1 (%)					
No	3178 (60.4)	1811 (62.7)	1361 (64.9)	1199 (65.3)	164 (64.1)
Yes	2081 (39.6)	1076 (37.3)	735 (35.1)	637 (34.7)	92 (35.9)
CKM diagnosis at year 5 (%)					
No	1688 (32.1)	—	—	—	—
Yes	1675 (31.9)	—	—	—	—
TYG-BMI	183.28±25.72	178.79±25.97	177.52±25.08	182.00±26.72	183.40±25.65
METS_IR	30.49±4.65	30.54±4.67	29.98±4.53	30.77±4.82	30.57±4.61
SPISE	6.91±1.41	7.03±1.50	7.12±1.45	6.87.10±1.44	6.87±1.38

BMI, body mass index; CKM, cardiovascular–kidney–metabolic; FBG, fasting blood glucose; TG, triglycerides; TC, total cholesterol; CR, creatinine; BUN, blood urea nitrogen; HDL-C, high-density lipoprotein cholesterol; LDL-C, low-density lipoprotein cholesterol; METS-IR, metabolic score for insulin resistance; SPISE, single-point insulin sensitivity estimator; TYG-BMI, triglyceride-glucose-body mass index.

Of the 5,259 participants enrolled in 2015, 3,363 (63.9%) had available 5-year outcome data and 1,896 (36.1%) were lost to follow-up.

Each columns represent baseline enrollment cohorts. One-year outcomes were assessed in the year following baseline; Five-year outcomes were assessed in 2020 for the 2015 cohort.

**Table 2** shows the associations between the IR indices and 1-year CKM syndrome incidence. The TyG–BMI and METS–IR were positively associated with 1-year CKM syndrome risk. Per 1 SD increase, the fully adjusted ORs were 1.55 (95% CI: 1.49–1.62) for TyG–BMI and 1.54 (95% CI: 1.47–1.60) for METS–IR (both *p* < 0.001). Compared with the lowest quartile, participants in the highest quartile had a 4.47-fold (TyG–BMI) and 4.06–fold (METS-IR) higher odds of developing CKM syndrome, with significant dose–response trends (*p* for trend < 0.001). SPISE showed an inverse association with CKM syndrome risk, with each SD increase associated with a 36% lower odds (OR = 0.64, 95% CI: 0.62–0.67, *p* < 0.001). Participants in Q4 had a 68% lower odds (OR = 0.32, 95% CI: 0.27–0.38) compared with those in Q1, with a significant dose–response relationship (*p* for trend < 0.001).

**Table 2:** Associations between insulin resistance indices and 1-year incidence of cardiovascular-kidney-metabolic syndrome.

Variable	Model 1			Model 2			Model 3			Model 4		
	OR (95% CI)	P-value	P_trend_	OR (95% CI)	P-value	P_trend_	OR (95% CI)	P-value	P_trend_	OR (95% CI)	P-value	P_trend_
TYG-BMI												
per SD	1.75 (1.69, 1.82)	<0.001		1.57 (1.51, 1.64)	<0.001		1.58 (1.51, 1.65)	<0.001		1.55 (1.49, 1.62)	<0.001	
Q1	Reference	—	<0.001	Reference	—	<0.001	Reference	—	<0.001	Reference	—	<0.001
Q2	1.71 (1.54, 1.90)	<0.001		1.53 (1.38, 1.71)	<0.001		1.53 (1.38, 1.71)	<0.001		1.48 (1.33, 1.66)	<0.001	
Q3	3.11 (2.79, 3.48)	<0.001		2.37 (2.10, 2.66)	<0.001		2.36 (2.10, 2.66)	<0.001		2.27 (2.01, 2.56)	<0.001	
Q4	6.43 (5.51, 7.52)	<0.001		4.65 (3.96, 5.48)	<0.001		4.72 (4.01, 5.56)	<0.001		4.47 (3.79, 5.28)	<0.001	
METS-IR												
per SD	1.66 (1.60, 1.73)	<0.001		1.50 (1.44, 1.57)	<0.001		1.51 (1.45, 1.57)	<0.001		1.54 (1.47, 1.60)	<0.001	
Q1	Reference	—	<0.001	Reference	—	<0.001	Reference	—	<0.001	Reference	—	<0.001
Q2	1.43 (1.29, 1.58)	<0.001		1.27 (1.15, 1.41)	<0.001		1.28 (1.15, 1.42)	<0.001		1.30 (1.17, 1.45)	<0.001	
Q3	2.67 (2.40, 2.98)	<0.001		2.08 (1.85, 2.33)	<0.001		2.09 (1.86, 2.34)	<0.001		2.18 (1.93, 2.45)	<0.001	
Q4	5.16 (4.44, 6.01)	<0.001		3.81 (3.25, 4.48)	<0.001		3.85 (3.28, 4.52)	<0.001		4.06 (3.45, 4.78)	<0.001	
SPISE												
per SD	0.58 (0.55, 0.60)	<0.001		0.64 (0.61, 0.67)	<0.001		0.64 (0.61, 0.67)	<0.001	<0.001	0.64 (0.62, 0.67)	<0.001	
Q1	Reference	—	<0.001	Reference	—	<0.001	Reference	—		Reference	—	<0.001
Q2	0.46 (0.42, 0.50)	<0.001		0.51 (0.47, 0.57)	<0.001		0.51 (0.46, 0.56)	<0.001		0.51 (0.47, 0.57)	<0.001	
Q3	0.27 (0.24, 0.30)	<0.001		0.35 (0.31, 0.39)	<0.001		0.35 (0.31, 0.39)	<0.001		0.35 (0.31, 0.40)	<0.001	
Q4	0.23 (0.20, 0.27)	<0.001		0.32 (0.27, 0.37)	<0.001		0.32 (0.27, 0.37)	<0.001		0.32 (0.27, 0.38)	<0.001	

OR, odds ratio; CI, confidence interval; SD, standard deviation; Q, quartile; METS-IR, metabolic score for insulin resistance; SPISE, single-point insulin sensitivity estimator; TYG-BMI, triglyceride-glucose-body mass index.

Model 1 adjusts no covariate. Model 2 adjusts age, sex and year. Model 3 further adjusts smoking and alcohol drinking frequency. Model 4 further adjusts blood pressure, lipid profile, and kidney function. .

**Figure 2:**
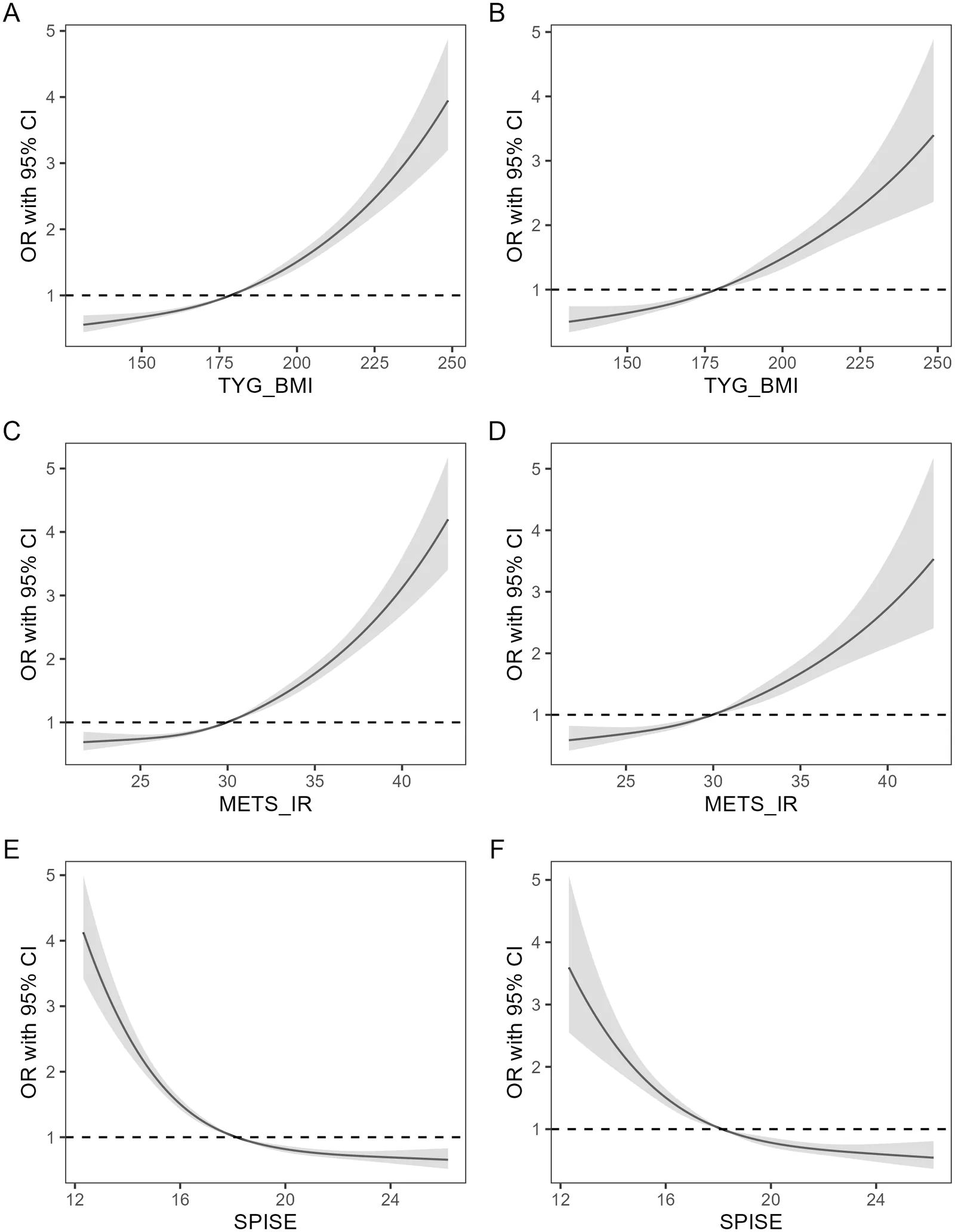
Nonlinear dose–response relationships between metabolic indices and CKM syndrome risk assessed by restricted cubic spline analysis. **(A)** Association between TyG_BMI and CKM syndrome risk at 1-year follow-up. **(B)** Association between TyG_BMI and CKM syndrome risk at 5-year follow-up. **(C)** Association between METS_IR and CKM syndrome risk at 1-year follow-up. **(D)** Association between METS_IR and CKM syndrome risk at 5-year follow-up. **(E)** Association between SPISE and CKM syndrome risk at 1-year follow-up. **(F)** Association between SPISE and CKM syndrome risk at 5-year follow-up. Solid lines indicate adjusted ORs; gray shaded areas represent 95% CIs. Dashed horizontal lines denote the null reference (OR = 1). Models were adjusted for age, sex, smoking status, alcohol status, blood pressure, lipid profiles, and kidney function. CKM, cardiovascular–kidney–metabolic; OR, odds ratio; CI, confidence interval; METS–IR, Metabolic Score for Insulin Response; SPISE, Single-Point Insulin Sensitivity Estimator; TyG–BMI, Triglyceride–Glucose–Body Mass Index.

**Table 3** shows the results of analysis of the associations between the IR indices and 5-year CKM syndrome incidence, which were consistent with those of the 1-year analysis. In the fully adjusted model (Model 4), TyG–BMI (OR per SD = 1.54, 95% CI: 1.44–1.66) and METS–IR (OR per SD = 1.51, 95% CI: 1.40–1.62) were positively associated with CKM syndrome risk, while SPISE (OR per SD = 0.64, 95% CI: 0.59–0.68) showed a protective association. Significant dose–response relationships were observed across all quartiles (*p* for trend < 0.001). The 5-year findings corroborate that IR is an independent predictor of CKM incidence over both the short- and long-term follow-up. RCS analyses were further performed to evaluate potential nonlinear associations between metabolic indices and CKM syndrome risk. After full adjustment for confounders, all three indices exhibited significant nonlinear dose-response relationships with CKM incidence (p for non-linearity < 0.05; **Figure 2**). To explore potential clinical application, optimal cut-off values were identified using the Youden index (**Supplementary Table 1**). The derived thresholds were close to the central distribution of the study population, with modest sensitivities and specificities (0.56–0.66).

**Table 3:** Associations between insulin resistance indices and 5-year incidence of cardiovascular-kidney-metabolic syndrome.

Variable	Model 1			Model 2			Model 3			Model 4		
	OR (95% CI)	P-value	P_trend_	OR (95% CI)	P-value	P_trend_	OR (95% CI)	P-value	P_trend_	OR (95% CI)	P-value	P_trend_
TYG-BMI												
per SD	1.83 (1.71, 1.95)	<0.001		1.56 (1.45, 1.67)	<0.001		1.56 (1.46, 1.68)	<0.001		1.54 (1.44, 1.66)	<0.001	
Q1	Reference	—	<0.001	Reference	—	<0.001	Reference	—	<0.001	Reference	—	<0.001
Q2	1.74 (1.47, 2.06)	<0.001		1.48 (1.24, 1.77)	<0.001		1.50 (1.25, 1.79)	<0.001		1.45 (1.21, 1.74)	<0.001	
Q3	3.46 (2.89, 4.14)	<0.001		2.39 (1.97, 2.90)	<0.001		2.40 (1.98, 2.92)	<0.001		2.30 (1.89, 2.81)	<0.001	
Q4	6.43 (4.95, 8.40)	<0.001		3.91 (2.97, 5.19)	<0.001		3.96 (3.00, 5.26)	<0.001		3.81 (2.87, 5.07)	<0.001	
METS-IR												
per SD	1.69 (1.58, 1.80)	<0.001		1.46 (1.36, 1.56)	<0.001		1.46 (1.37, 1.57)	<0.001		1.51 (1.40, 1.62)	<0.001	
Q1	Reference	—	<0.001	Reference	—	<0.001	Reference	—	<0.001	Reference	—	<0.001
Q2	1.68 (1.44, 1.96)	<0.001		1.43 (1.22, 1.69)	<0.001		1.46 (1.24, 1.72)	<0.001		1.52 (1.29, 1.80)	<0.001	
Q3	3.03 (2.56, 3.60)	<0.001		2.16 (1.80, 2.60)	<0.001		2.19 (1.82, 2.63)	<0.001		2.34 (1.94, 2.83)	<0.001	
Q4	4.99 (3.85, 6.52)	<0.001		3.17 (2.40, 4.21)	<0.001		3.22 (2.44, 4.29)	<0.001		3.55 (2.67, 4.74)	<0.001	
SPISE												
per SD	0.55 (0.51, 0.59)	<0.001		0.64 (0.59, 0.68)	<0.001		0.64 (0.59, 0.68)	<0.001		0.64 (0.59, 0.68)	<0.001	
Q1	Reference	—	<0.001	Reference	—	<0.001	Reference	—	<0.001	Reference	—	<0.001
Q2	0.46 (0.40, 0.54)	<0.001		0.55 (0.46, 0.64)	<0.001		0.55 (0.46, 0.65)	<0.001		0.54 (0.46, 0.64)	<0.001	
Q3	0.24 (0.20, 0.28)	<0.001		0.33 (0.27, 0.40)	<0.001		0.33 (0.27, 0.39)	<0.001		0.32 (0.27, 0.39)	<0.001	
Q4	0.19 (0.15, 0.25)	<0.001		0.29 (0.22, 0.39)	<0.001		0.29 (0.22, 0.39)	<0.001		0.30 (0.23, 0.40)	<0.001	

OR, odds ratio; CI, confidence interval; SD, standard deviation; Q, quartile; METS-IR, metabolic score for insulin resistance; SPISE, single-point insulin sensitivity estimator; TYG-BMI, triglyceride-glucose-body mass index.

Model 1 adjusts no covariate. Model 2 adjusts age, sex and year. Model 3 further adjusts smoking and alcohol drinking frequency. Model 4 further adjusts blood pressure, lipid profile, and kidney function. .

**Table 4** presents the discriminatory performance of Model 0 and the models formed by separately adding each IR index for predicting 1- and 5-year CKM syndrome risk. For 1-year CKM prediction, the base model without IR indices (Model 0) achieved an AUROC of 0.681. Adding TyG-BMI significantly improved discrimination to 0.710 (p < 0.001). Similarly, METS-IR (AUROC = 0.710) and SPISE (AUROC = 0.712) also demonstrated significant improvements over Model 0. For 5-year CKM prediction, Model 0 yielded an AUROC of 0.719. All three IR indices significantly enhanced predictive performance: TyG-BMI (AUROC = 0.739), METS-IR (AUROC = 0.737), and SPISE (AUROC = 0.741). Pairwise DeLong tests were performed to compare the three indices directly. SPISE showed statistically higher AUROCs than METS-IR at both 1 year (P = 0.006) and 5 years (P = 0.001), and then TyG-BMI at 1 year (P = 0.010). However, the absolute differences were small (0.002–0.003), indicating broadly comparable predictive performance (**Supplementary Table 2**).

**Table 4:** Comparison of predictive performance for CKM syndrome with and without insulin resistance indices.

Variable	1-year CKM			5-year CKM		
	Model 1	Model 0	P	Model 1	Model 0	P
TYG-BMI	0.710 (0.701, 0.720)	0.681 (0.672, 0.691)	<0.001	0.739 (0.725, 0.753)	0.719 (0.705, 0.734)	<0.001
METS-IR	0.710 (0.700, 0.719)		<0.001	0.737 (0.723, 0.752)		<0.001
SPISE	0.712 (0.703, 0.722)		<0.001	0.741 (0.727, 0.755)		<0.001

CKM, cardiovascular-kidney-metabolic; METS-IR, metabolic score for insulin resistance; SPISE, single-point insulin sensitivity estimator; TYG-BMI, triglyceride-glucose-body mass index.

Model 1 includes the insulin resistance index, sex, age, year, smoking, alcohol, blood pressure, lipid profile and kidney function. Model 0 is the base model and includes sex, age, year, smoking, alcohol, blood pressure, lipid profile and kidney function.

**Table 5** shows the results of subgroup analyses of the IR indices and 1-year and 5-year CKM syndrome risk by age, sex, and BMI. For 1-year risk, significant interactions were observed for age (all *p* < 0.001), sex (all *p* < 0.05), and BMI (SPISE: *p* < 0.001). The associations between the IR indices and CKM syndrome risk were consistent across age groups, with participants < 40 years showing slightly stronger associations compared with those ≥ 40 years. Sex-based differences were pronounced, with males demonstrating stronger associations. BMI significantly modified the SPISE association, showing a stronger protective effect in participants with BMI ≥ 19. For 5-year risk, significant sex-based interactions were observed for all three indices (all *p* < 0.001), with males showing stronger associations compared with females. No significant interactions were detected for age or BMI in the 5-year analysis.

**Table 5:** Subgroup analysis of associations between insulin resistance indices and 1-year and 5-year CKM incidence.

Subgroup	TYG-BMI		METS-IR		SPISE	
	OR (95% CI)	P_interaction_	OR (95% CI)	P_interaction_	OR (95% CI)	P_interaction_
1-year CKM risk						
Age						
≥40	1.48 (1.41, 1.56)	<0.001	1.47 (1.40, 1.54)	<0.001	0.67 (0.64, 0.70)	<0.001
<40	1.66 (1.59, 1.74)		1.67 (1.59, 1.75)		0.60 (0.57, 0.62)	
Sex						
Male	1.61 (1.53, 1.69)	0.010	1.64 (1.56, 1.72)	<0.001	0.59 (0.56, 0.62)	<0.001
Female	1.47 (1.40, 1.55)		1.43 (1.36, 1.50)		0.69 (0.66, 0.72)	
BMI						
≥19	1.62 (1.56, 1.68)	0.662	1.61 (1.55, 1.68)	0.309	0.59 (0.57, 0.62)	<0.001
<19	1.74 (1.24, 2.44)		1.36 (0.97, 1.89)		0.82 (0.70, 0.97)	
5-year CKM risk						
Age						
≥40	1.56 (1.40, 1.74)	0.572	1.53 (1.37, 1.70)	0.443	0.65 (0.58, 0.72)	0.327
<40	1.63 (1.48, 1.80)		1.61 (1.46, 1.78)		0.60 (0.55, 0.66)	
Sex						
Male	1.82 (1.63, 2.05)	<0.001	1.77 (1.59, 1.98)	<0.001	0.54 (0.48, 0.61)	<0.001
Female	1.41 (1.28, 1.55)		1.38 (1.25, 1.52)		0.71 (0.64, 0.77)	
BMI						
≥19	1.64 (1.50, 1.78)	0.980	1.60 (1.47, 1.74)	0.800	0.59 (0.54, 0.64)	0.149
<19	1.62 (0.82, 3.20)		1.47 (0.76, 2.84)		0.76 (0.54, 1.07)	

CKM, cardiovascular-kidney-metabolic; SD, standard deviation; OR, odds ratio; CI, confidence interval; METS-IR, metabolic score for insulin resistance; SPISE, single-point insulin sensitivity estimator; TYG-BMI, triglyceride-glucose-body mass index; BMI, body mass index. .

## 4 Discussion

In this large-scale retrospective cohort study, we assessed the relationship between three IR indices—METS–IR, TyG–BMI, and SPISE—and the risk of CKM syndrome. After finding that all three indices were independently associated with the risk of developing CKM syndrome, we conducted RCS analysis, which showed that these three indicators had a non-linear relationship with CKM syndrome risk. Moreover, the three indices showed broadly comparable discriminatory performance, with SPISE yielding slightly higher AUROCs at both follow-up points. Further subgroup analysis demonstrated that all three indices were more strongly correlated with the risk of CKM syndrome in males compared with females.

A recent study that evaluated the relationship between IR and the prevalence of CKM syndrome showed that the homeostatic model assessment for insulin resistance (HOMA-IR) and the homeostatic model assessment of beta-cell function (HOMA-β) are positively correlated with the occurrence of advanced CKM syndrome in Chinese adults with type 2 diabetes mellitus (T2DM) (27). Another study demonstrated that the HOMA-IR is positively correlated with the mortality rate of patients with CKM syndrome (28). Although the HOMA-IR and HOMA-β have traditionally been used as basic measurement tools for IR due to their low practicality and variability, these indicators are not suitable for large-scale studies.

As simple and easily accessible markers for IR, the TyG–BMI, METS–IR, and SPISE have been validated in different populations. Wu et al. demonstrated that in patients with CKM syndrome, elevated METS–IR levels are associated with an increased risk of stroke (29). Li et al. proposed that the TyG–BMI index has a positive linear correlation with increased CVD incidence in patients with CKM syndrome (22). A cross-sectional study showed that the SPISE can serve as a long-term risk predictor for coronary heart disease and T2DM (30). However, before the current study, no research had investigated the predictive value of the TyG–BMI, METS–IR, and SPISE for the risk of CKM syndrome simultaneously. This large-scale, community-based retrospective cohort study was the first to demonstrate that the TyG–BMI, METS–IR, and SPISE are independently associated with the occurrence of CKM syndrome. Compared with their predictive value for the occurrence of CKM syndrome in the short (1-year) term, these three indicators have much stronger predictive value for the risk of CKM syndrome in the long (5-year) term. Thus, they have a stable predictive ability for the occurrence of CKM syndrome.

These three indicators can also be used to predict the mechanism of CKM syndrome, which is currently unknown. Several potential mechanisms may explain the relationship between these three indicators and the risk of CKM syndrome, a systemic disease characterized by pathological physiological interactions among metabolic risk factors, CKD, and the cardiovascular system. During early CKM syndrome (Stages 0–1), IR promotes the progression of CKM syndrome by causing adipose tissue dysfunction and lipid imbalance, which mainly manifest as elevated TG levels, decreased HDL-C levels, and increased low-density lipoprotein particles. Under the combined effect of these factors and pro-inflammatory cytokines, such as IL-6 and TNF-α, the development of atherosclerosis accelerates (31). As CKM syndrome progresses to Stages 2–3, IR increases the risk of further development by fostering endothelial dysfunction. The main manifestation of this dysfunction is a reduction in the bioavailability of nitric oxide, which hinders vasodilation and promotes hypertension and ventricular remodeling (32). IR also promotes metabolic disorders, leading to vascular inflammation and atherosclerosis, thereby accelerating the occurrence and development of CKM syndrome.

Among the three IR indicators, the SPISE demonstrated higher AUC values, suggesting that it tends to show better predictive performance for CKM syndrome incidence in both the short- and long-term relative to the other two IR indicators. This finding is consistent with a Spanish study, which found that the SPISE demonstrated a superior ability to predict cardiometabolic risk in adolescents compared with traditional indicators such as the HOMA–IR, TG/HDL-C ratio, and TyG index (14). A study assessing the relationship between the SPISE and the risk of metabolic syndrome in the Korean population also observed that the predictive ability of the SPISE for metabolic syndrome was superior to that of the HOMA-IR, inverse insulin, TG/HDL-C ratio, and TyG index (19). Taken together, these findings suggest that, compared with other markers of IR, SPISE may show slightly greater predictive ability for CKM syndrome risk and offer potential clinical utility for early risk identification in the Chinese population.

From a practical perspective, all three indices can be readily calculated from routinely collected anthropometric and laboratory measurements. In this cohort, the cut-off values for METS-IR, TyG-BMI, and SPISE were 30.89, 182.65, and 6.72, respectively, for 1-year CKM syndrome risk and 30.75, 183.31, and 6.81, respectively, for 5-year risk. These cohort-derived cut-off values showed a reasonable balance between sensitivity and specificity, supporting their potential use as reference points for initial CKM risk stratification in community settings. Incorporating these indices into routine health assessments may help identify individuals who would benefit from more comprehensive evaluation. External validation is needed to confirm the generalizability of these cut-off values and support their broader application.

Subgroup analysis revealed that compared with the female population, the correlation of these three indicators with CKM syndrome was more significant in the male population. In accordance, Wani et al. discovered that the gender-specific cut-offs for the SPISE in predicting metabolic syndrome in Arab adolescents were ≤6.1 for males and ≤6.46 for females (33). Another study demonstrated that the SPISE performed significantly better in identifying metabolic syndrome and IR in males compared with females (34). Likewise, a study of cardiovascular and metabolic risk in adolescents found that the SPISE had higher diagnostic accuracy in males than in females (14). These findings, consistent with our research findings, reflect the different diagnostic performance of the SPISE for males and females. However, as the research subjects in the other studies were adolescents, their findings cannot be generalized to adults. Expanding our research sample to include adults, we confirmed the existence of sex differences in the predictive power of the SPISE for CKM syndrome. The cause of sex differences might be related to the fact that men accumulate more visceral and liver fat than women (35), which could lead to higher IR in the male population. Previous studies have also found that the prevalence of advanced CKM syndrome stage is higher in men than in women (36). By confirming this association, our research adds to the knowledge base on the prevention of CKM syndrome in males.

Our regional representative retrospective cohort study had several methodological advantages that add to the reliability and validity of the findings. First, we evaluated the predictive value of three IR indicators for the risk of CKM syndrome in the Chinese population, providing new insights for the early identification and prevention of this disease. Second, we used RCS analysis to assess the nonlinear relationship between the three IR indicators and the risk of CKM syndrome, thereby enhancing the reliability of the results. Finally, during the analysis process, we controlled for potential confounding factors as much as possible and conducted subgroup analyses to identify differences in the prognostic value of the indicators in subgroups, providing a new perspective for the stratified management of CKM syndrome. However, several limitations deserve to be considered. Our use of an observational design precluded definitive causal inference, as residual confounders may have persisted despite rigorous adjustment for a wide range of established and potential confounders. All the participants came from a single center. The lack of multi-center participation inevitably compromises the representativeness of the study population and thereby restricts the extrapolation of our findings to other geographical regions or ethnic populations. We assessed the predictive performance of the three IR indices only in this study and did not seek independent external multi-center validation, which may have introduced overfitting. Therefore, any firm conclusion regarding the reproducibility and stability of the observed associations across heterogeneous clinical settings may be precluded. The ascertainment of CKM syndrome was partially derived from self-reported medical history rather than by independent physician assessment through medical record review or by standardized clinical assessment. Self-report methodology is inherently prone to recall bias, and the consequent non-differential misclassification of the outcome would be expected to bias the effect estimates toward the null, thereby potentially underestimating the true strength of the associations. The present analysis used baseline measurements and did not account for changes in lifestyle factors, clinical characteristics, or IR indices during follow-up. Future studies incorporating repeated measurements and time-varying analyses could clarify their associations with CKM stage progression. Additionally, these IR indices were associated with CKM syndrome risk and may improve discrimination when considered alongside the component variables, the extent to which this improvement reflects the composite index itself versus its constituent variables requires further investigation.

## 5 Conclusion

In this community-based cohort, the METS-IR, TyG-BMI, and SPISE showed robust nonlinear associations with 1- and 5-year CKM syndrome risk. Their discriminatory performance was broadly comparable, with SPISE showing a slight advantage. These associations were consistently stronger in males, supporting the integration of these readily available indices into sex-tailored early screening strategies for early CKM risk stratification in community health assessments.

## Data Availability

The data analyzed in this study is subject to the following licenses/restrictions: All data generated or analyzed during this study are included in this article. Further enquiries can be directed to the corresponding author. Requests to access these datasets should be directed to XY yinxianhong@yeah.net.
