# Supplementary material for: Short- and long-term associations of insulin resistance indices with cardiovascular–kidney–metabolic syndrome risk: a retrospective cohort study
Source: Front Endocrinol (Lausanne). 2026 Aug 31;17:1917797. doi: 10.3389/fendo.2026.1917797 (PMC13572117; doi:10.3389/fendo.2026.1917797)
Supplement: Supplementary file 1 [file DataSheet1.docx]

Study Population and Missing Data

Participants free of CKM at baseline were included in the analysis. Missing data were handled using complete-case analysis, with only participants with complete data for all variables of interest included in the final analyses.

Insulin Resistance Indices

Three metabolic exposure indices were analyzed: triglyceride-glucose body mass index (TYG-BMI), metabolic score for insulin resistance (METS-IR), and single-point insulin sensitivity estimator (SPISE). Continuous exposure variables were standardized using Z-score transformation and truncated at the 1st and 99th percentiles to minimize the influence of outliers. For categorical analyses, exposures were divided into quartiles (Q1–Q4), with Q1 (lowest quartile) serving as the reference category. The linear trend across quartiles was evaluated by treating quartile rank as an ordinal variable.

Logistic Regression Analysis

Logistic regression models were fitted to assess the association between each metabolic exposure index and the risk of CKM at 1-year and 5-year follow-up. Four nested models with progressive covariate adjustment were constructed:

Model 1 (Unadjusted): exposure variable only

Model 2 (Demographic-adjusted): age, sex, and examination year

Model 3 (Lifestyle-adjusted): Model 2 covariates plus smoking status and alcohol consumption frequency

Model 4 (Fully adjusted): Model 3 covariates plus systolic blood pressure, diastolic blood pressure, total cholesterol, triglycerides, high-density lipoprotein cholesterol, low-density lipoprotein cholesterol, fasting blood glucose, creatinine, and blood urea nitrogen

Results were expressed as odds ratios (ORs) with 95% confidence intervals (CIs) per 1-SD increase in the standardized exposure. All P-values were two-sided, and P < 0.05 was considered statistically significant.

Non-linear Relationship Analysis

Restricted cubic spline (RCS) regression with 4 knots was employed to explore potential non-linear relationships between each metabolic exposure index and CKM risk. The RCS model was fitted using the fully adjusted strategy (Model 4), allowing for flexible modeling of the exposure-outcome relationship.

Model Discriminatory Performance

The discriminatory performance of each predictive model was evaluated using the area under the receiver operating characteristic curve (AUROC). Two nested logistic regression models were constructed: Model 0 (Reference), containing covariates only (sex, age, year, smoking, alcohol, blood pressure, lipid profile, and kidney function); and Model 1 (Full), containing Model 0 covariates plus the metabolic exposure index (TYG-BMI, METS-IR, or SPISE). Restricted cubic splines with 4 knots were applied to model the exposure variable in Model 1, allowing for potential non-linear relationships with outcomes. The statistical significance of the difference in AUROC between Model 1 and Model 0 was assessed using the DeLong test.

Subgroup Analysis and Interaction Testing

Subgroup analyses were conducted to examine the effect modifications of age, sex, and BMI on the associations between insulin resistance indices and CKM incidence at 1-year and 5-year follow-up. Stratified analyses were performed within each subgroup (age: ≥40 and <40 years; sex: male and female; BMI: ≥19 and <19 kg/m²), with risk estimates calculated per 1-SD increase in each insulin resistance index using the fully adjusted model (Model 4). Interaction effects were statistically tested by adding a multiplicative interaction term between the insulin resistance index and the stratification variable into the logistic regression model, adjusting for all covariates as previously described. The significance of the interaction was evaluated using the likelihood ratio test, with *P* for interaction < 0.05 considered statistically significant.
