# Supplementary material for: Short- and long-term associations of insulin resistance indices with cardiovascular–kidney–metabolic syndrome risk: a retrospective cohort study
Source: Front Endocrinol (Lausanne). 2026 Aug 31;17:1917797. doi: 10.3389/fendo.2026.1917797 (PMC13572117; doi:10.3389/fendo.2026.1917797)
Supplement: Supplementary file 2 [file Table1.docx]

| Table S1. Optimal cut-off values and predictive performance of metabolic indices for 1-year and 5-year CKM risk based on Youden index. | | | | | |
| --- | --- | --- | --- | --- | --- |
| Index | Cut-off value | Sensitivity | Specificity | Youden index | AUROC |
| 1-year CKM risk | |  |  |  |  |
| TYG_BMI | 182.65 | 0.63 | 0.60 | 0.23 | 0.65 (0.65-0.66) |
| METS_IR | 30.89 | 0.60 | 0.61 | 0.21 | 0.64 (0.63-0.65) |
| SPISE | 6.72 | 0.65 | 0.57 | 0.23 | 0.65 (0.65-0.66) |
| 5-year CKM risk |  |  |  |  |  |
| TYG_BMI | 183.31 | 0.60 | 0.65 | 0.25 | 0.66 (0.65-0.68) |
| METS_IR | 30.75 | 0.56 | 0.66 | 0.21 | 0.64 (0.63-0.66) |
| SPISE | 6.81 | 0.63 | 0.60 | 0.23 | 0.66 (0.64-0.67) |
| Abbreviations: CKM, cardiovascular-kidney-metabolic; METS-IR, metabolic score for insulin resistance; SPISE, single-point insulin sensitivity estimator; TYG-BMI, triglyceride-glucose-body mass index; AUROC, area of receiver operating characteristics curve. | | | | | |
