# Supplementary material for: Short- and long-term associations of insulin resistance indices with cardiovascular–kidney–metabolic syndrome risk: a retrospective cohort study
Source: Front Endocrinol (Lausanne). 2026 Aug 31;17:1917797. doi: 10.3389/fendo.2026.1917797 (PMC13572117; doi:10.3389/fendo.2026.1917797)
Supplement: Supplementary file 3 [file Table2.docx]

| Table S2. Pairwise comparison of AUROC for metabolic indices in predicting 1-year and 5-year CKM risk using DeLong test. | | | | |
| --- | --- | --- | --- | --- |
| Index 1 | Index 2 | AUROC 1 | AUROC 2 | P-value |
| 1-year CKM risk | |  |  |  |
| TYG_BMI | METS_IR | 0.710 (0.701-0.720) | 0.710 (0.701-0.719) | 0.809 |
| TYG_BMI | SPISE | 0.710 (0.701-0.720) | 0.712 (0.703-0.722) | 0.010 |
| METS_IR | SPISE | 0.710 (0.701-0.719) | 0.712 (0.703-0.722) | 0.006 |
| 5-year CKM risk | |  |  |  |
| TYG_BMI | METS_IR | 0.739 (0.725-0.753) | 0.738 (0.723-0.752) | 0.271 |
| TYG_BMI | SPISE | 0.739 (0.725-0.753) | 0.741 (0.727-0.755) | 0.033 |
| METS_IR | SPISE | 0.738 (0.723-0.752) | 0.741 (0.727-0.755) | 0.001 |
| Abbreviations: CKM, cardiovascular-kidney-metabolic; METS-IR, metabolic score for insulin resistance; SPISE, single-point insulin sensitivity estimator; TYG-BMI, triglyceride-glucose-body mass index; AUROC, area of receiver operating characteristics curve. | | | | |
